# VX‐765 alleviates motor and cognitive impairments via inhibiting PANoptosis activation in the neonatal rats after hypoxic–ischemic brain damage

**DOI:** 10.1002/pdi3.66

**Published:** 2024-05-01

**Authors:** Xiaohuan Li, Mulan Chen, Boqing Xu, Yepeng Fan, Chunfang Dai, Zhifang Dong

**Affiliations:** ^1^ Growth, Development, and Mental Health of Children and Adolescence Center Pediatric Research Institute Ministry of Education Key Laboratory of Child Development and Disorders National Clinical Research Center for Child Health and Disorders Chongqing Key Laboratory of Child Neurodevelopment and Cognitive Disorders Children's Hospital of Chongqing Medical University Chongqing China; ^2^ Department of Children Health Care Guangzhou Women and Children's Medical Center Guangzhou Medical University Guangzhou Guangdong China

**Keywords:** caspase‐1, hypoxic‐ischemic encephalopathy, neuroinflammation, PANoptosis, VX‐765

## Abstract

Neonatal hypoxia–ischemia (HI) is one of the main factors that cause neonatal severe neurologic impairment and death. Shown by a large number of studies, caspase‐1 plays a significant effect in diseases such as hypoxic–ischemic brain damage (HIBD) and may be a key component of the protein complex that initiates PANoptosis. VX‐765, an inhibitor of caspase‐1, exerts a potential neuroprotective effect in traumatic brain injury. However, it is unknown whether the administration of VX‐765 has neuroprotective effects on neonatal rats that suffered HIBD, and if so, the underlying mechanisms are also still unknown. In the present study, we found that treatment with VX‐765 (50 mg/kg, i.p.) significantly ameliorated the impairment of locomotor coordination functions and myodynamia as well as the spatial learning and memory in neonatal rats subjected with HIBD. These behavior improvements were attributed to VX‐765 reducing infarct volumes and neuronal loss in the CA1, CA3 region of hippocampus, and deeper layers of the cortex in HIBD rats. Moreover, the enzyme‐linked immunosorbent assay showed that VX‐765 obviously decreased the production of neuroinflammatory factors including TNF‐α, IL‐1β, and IL‐6. Importantly, we identified HI promoted PANoptosis activation in vivo and in vitro, and VX‐765 obviously suppressed PANoptosis activation. Finally, we demonstrated that VX‐765 treatment reversed neuronal injury induced by oxygen–glucose deprivation (OGD). Taken together, these results suggest that VX‐765 protects the neurons against damage by suppressing neuroinflammation and PANoptosis activation, thereby improving locomotor coordination and cognitive impairments in neonatal HIBD rats, indicating that VX‐765 may be an underlying therapeutic drug for the clinical treatment of hypoxic–ischemic encephalopathy (HIE).

## Graphical abstract

The inflammation cytokines is increased and the PANoptosis is activated after hypoxia–ischemia (HI). And VX‐765 suppressing neuroinflammation release and PANoptosis activation, thereby improving motor and cognitive impairments induced by HI in the neonatal rats.

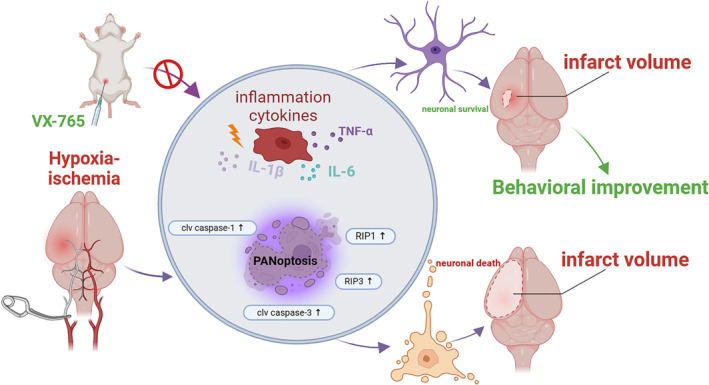

## 1 INTRODUCTION

Perinatal asphyxia induces a series of clinical manifestations, which seriously affects the health of newborns. HIE, caused by perinatal asphyxia, is the most prevalent and severe type for it can induce neonatal brain injury.^1^ HIE is the second most important factor of neonatal death after complications of premature delivery and accounts for about 25% of neonatal mortality.^2^, ^3^ Previous reports showed that the prevalence rate of HIE which causes long‐term cognitive impairment of children was 1.5‰.^1^ Patients with severe HIE often suffer from sequelae such as cerebral palsy, epilepsy, and learning impairment.^4^, ^5^, ^6^ However, mild hypothermia therapy, one of the most commonly used therapies for HIE, is so far not effective for all patients.^7^ Developing novel treatment methods to improve therapeutic efficacy becomes urgent.

There is evidence that apoptosis and pyroptosis occur during and/or after brain HI,^5^, ^6^ and when the apoptotic signaling is amplified, then the affected cells lead to necroptosis.^8^, ^9^ Furthermore, substantial research indicates that programmed cell death pathways, pyroptosis, apoptosis, and necroptosis play a pivotal role in HIE. Programmed cell death pathways are governed by distinctive proteins that orchestrate a multitude of biological consequences.^8^, ^9^, ^10^, ^11^, ^12^ Pyroptosis, apoptosis, and necroptosis have extensive cross talk, which leads to the concept of “PANoptosis.” PANoptosis is a unique inflammatory programmed cell death, which allows for interactions and activation of the machinery required for inflammasome/pyroptosis (caspase‐1), apoptosis (caspase‐3), and necroptosis (RIP1/RIP3). PANoptosis plays a crucial role in several diseases, including infectious diseases, autoinflammatory diseases, and cancer.^13^, ^14^, ^15^, ^16^, ^17^, ^18^ The understanding and manipulation of this pathway could potentially lead to new therapeutic strategies for these diseases. However, when PANoptosis is activated, blocking just one arm (i.e., blocking just pyroptosis or apoptosis or necroptosis alone) cannot prevent cell death or inflammatory cytokine‐induced diseases.^16^, ^19^ Several studies have suggested that the inflammasome/pyroptosis (caspase‐1) may be a critical component of the protein complex that initiates PANoptosis, the PANoptosome.^10^, ^20^

Caspase‐1 is a form of cysteine proteases encoded by the sequence of 11q22.1 in humans as a key mediator of inflammatory responses widely expressed in the brain.^21^, ^22^ In the central nervous system (CNS), caspase‐1 has been implicated in various neurological disorders, including stroke, Alzheimer's disease, Parkinson's disease, and multiple sclerosis.^23^, ^24^ In these disorders, caspase‐1 contributes to the inflammatory response and cell death by inducing pyroptosis and the releasing of cytokines. Growing evidence has demonstrated that the caspase‐1 inflammasome‐signaling pathway is significantly activated in brain injuries after HI.^25^, ^26^, ^27^, ^28^ It has been reported that the expression of NLRP3, caspase‐1, and IL‐1 is enhanced in HI patients and animal models. Previous studies in caspase‐1 dominant‐negative transgenic or caspase‐1‐deficient mice have provided for the role of caspase‐1 in neuronal death and inflammatory pathways, such as caspase‐1‐deficient mice are protected against neuronal death and behavioral deficits after a stroke.^28^, ^29^, ^30^, ^31^ As the aforementioned, since pyroptosis, apoptosis, and necroptosis play a pivotal role in HIE, the cross talk of those three forms of cell death leads to the concept of PANoptosis, and caspase‐1 may be a critical component of the protein complex that initiates PANoptosis; we speculate that inhibiting caspase‐1 could simultaneously suppress those three forms of cell death, namely PANoptosis, thereby protecting neurons against damage caused by HI.

VX‐765 is a selective and developed small‐molecule inhibitor of caspase‐1, which has been demonstrated in various inflammatory disease models by inhibiting pro‐caspase‐1 self‐cleavage.^24^, ^31^, ^32^, ^33^, ^34^ VX‐765 is nontoxic and bioavailable and has been proved to be safe for humans by oral administration in epilepsy clinical trial studies.^23^, ^35^, ^36^, ^37^ There is evidence that VX‐765 treatment protects against traumatic brain injury by inhibiting pyroptosis.^23^, ^38^ However, whether VX‐765 inhibits the neuronal death and inflammatory pathways in neonatal rats after HIBD remains largely unknown. So here, we hypothesize that VX‐765 could present neuroprotective effects by suppressing neuroinflammation and PANoptosis activation in rats after HIBD. To demonstrate this hypothesis, we performed experiments both in vivo and in vitro in HI models.

## 2 MATERIALS AND METHODS

### 2.1 Animals

HI modeling surgery was performed on postnatal day 7 (P7) of Sprague‐Dawley (SD) rats to establish the HIBD model. Specifically, the left common carotid artery was ligated. Two hours after the surgery, the rats were placed in a closed container with 8% O_2_ and 92% N_2_ maintained for 2.5 h for hypoxia.^39^ The Sham group, rats only left common carotid artery isolation was performed and hypoxia treatment was not performed. The pups were returned to their home cage with dams after processing. In the present study, we used a total of 15 adult rats for breeding purposes and obtained about 100 rat pups from these pairings and 5 pregnant rats for culturing primary neurons. All animal experiments were performed in accordance with the Chongqing Science and Technology Commission guidelines and were approved by the Animal Ethics Committee of Children's Hospital of Chongqing Medical University (No. CHCMU‐IACUC20210114017).

### 2.2 Antibodies and reagents

Rabbit anti‐caspase‐1 (#ab1872) was supplied by Abcam Inc (Cambridge, MA, USA). Rabbit anti‐caspase‐3 (#9962S), rabbit anti‐RIP1 (#3493), and rabbit anti‐RIP3 (#95702) were purchased from Cell Signaling Technology (Danvers, MA, USA). Mouse anti‐β‐actin (#A5441) was supplied by Sigma‐Aldrich (St. Louis, MO, USA). Mouse anti‐NeuN (#MAB377) was obtained from Millipore Corporation (Billerica, MA, USA). LDH cytotoxicity assay kit was obtained from Beyotime Biotechnology (Shanghai, China). Rat TNF‐α ELISA Kit (#CSB‐E11987r), rat IL‐6 ELISA Kit (#CSB‐E04640r), and rat IL‐1β ELISA Kit (#CSB‐E08055r) were purchased from CUSABIO (Beijing, China). Annexin V‐FITC/PI apoptosis detection kit (#FXP018) was purchased from 4A BIOTECH (Suzhou, China). VX‐765 (#S2228) was purchased from Selleck Chemicals LLC (Houston, TX, USA).

### 2.3 Primary culture of neurons

A 19‐day‐old SD fetal rat was used to culture neurons as described previously with modification.^39^ Briefly, a pregnant rat was anaesthetized deeply with urethane solution (1.5 g/kg, i.p.) and then the fetal rat brain was isolated under sterile conditions. The cortex and hippocampus were isolated in a hank's balanced salt solution dissection buffer, then transferred to 3 mL of pre‐warmed 0.25% trypsin ethylenediaminetetraacetic acid to digest for 8 min in an incubator (Thermo Forma 3111, Thermo Scientific) at 37°C. After that, the dissociated cells were washed with Dulbecco's modified Eagle's medium (DMEM) (containing10% FBS) for 3 times and pipetted gently to get cell suspension. Then, the cell suspension was centrifuged at 600 rpm for 3 min, after re‐suspension in DMEM (containing 10% FBS) media, the cells were counted using a hemocytometer and plated onto poly‐D‐lysine pre‐treated culture dishes at a density of 1.0 × 10^7^ per 10 cm dish, 6.0 × 10^6^ per 6 cm dish, and 2.0 × 10^6^ per 6 well plate. The neurons were cultured in an incubator containing 5% CO_2_ at 37°C. 4–24 h later, the original media was replaced by Neurobasal Feeding (NF) Media (NF Media containing 500 mL of Neurobasal Media, 2% B27 supplement, and 1.25 mL GlutaMAX^TM^‐I Supplement). Half of NF Media was replaced every 3 days until the neurons were used for experiments.

### 2.4 OGD treatment

Seven days in vitro (DIV) primary cultured neurons were used in this study and the method of primary cultured neurons treated with OGD as previously described, with modifications.^39^ Briefly, the neurons conditioned medium (taken out and saved for further use) was replaced by Earle's balanced salt solution (EBSS) (#SH30253.01, HyClone, Utah, USA). And then, the neurons were transferred to an incubator (Thermo Scientific) in hypoxic conditions (5%O_2_ + 95%N_2_) at 37°C for 90 min. Then, the neurons were returned to the previously saved conditional medium. The neurons were then allowed to recover for 6 h until further experiments.

### 2.5 VX‐765 treatment

To explore the protective effects of VX‐765 in neurons treated with OGD, primarily cultured neurons were pretreated with VX‐765 (25 μM) for 1 h before OGD as previously described, with modifications.^40^ To explore whether VX‐765 presents protective effects on HIBD rats, VX‐765 was administered intraperitoneally (50 mg/kg, i.p.) for 1 h before HIBD surgery, and then treated daily for 7 days.^41^, ^42^, ^43^

### 2.6 Grasping test

Myodynamia of rat forelimbs was detected by a Grip Tester as previously reported, with modification.^39^ The left and right forelimbs of each rat (21 days after HIBD model established) were placed on the grip detector respectively. Their tails were gently pulled and the maximum tensile force was recorded until the rats could not hold on. The left or right forelimb myodynamia was measured five times and the mean value was calculated to express the myodynamia. The Grip Tester was cleaned with 70% ethanol between tests.

### 2.7 Rotarod test

Locomotor condition of rats was assessed on the rotarod after the griping test. Specifically, rats were put on the rotarod twice (rotary speed is 0 or 20 rpm, respectively) 1 day before formal tests as a pre‐training session. Ten trials (cut‐off time: 3 min) with 30 min of interval were given to the rats on the following day, in which speed accelerated from 5 to 50 rpm and the latency to fall off the rotarod was recorded to illustrate the motor performance. The rotarod was cleaned with 70% ethanol between tests.

### 2.8 Morris water maze test

To assess the spatial learning and memory functions of rats, Morris water maze was used as previously reported with modifications.^44^ Briefly, the water maze consists of a black circular pool (180 cm diameter, 60 cm height) filled with water (22 ± 1°C) containing nontoxic black dye to the level of 40 cm. The pool was divided into four equal quadrants designated as Northeast (NE), Southeast (SE), Southwest (SW), and Northwest (NW), and a platform (12 cm diameter) was placed 1 cm below the water surface at the SW quadrant. The rats (P35) were put into the pool for 60 s from the midpoint of NW on the first day to adapt to the water maze, trained to navigate and find the submerged platform for 4 trials per day within the next 5 days (day 1 to day 5). The rats were guided to the platform and allowed to stay on it for 10 s if they failed to find the platform during the training (cut‐off time: 60 s). Then, the platform was removed after a 5‐day training and a probe test was performed. The number of times that rats crossed the platform position, the time they spent to arrive at the previous platform position, and the total time they stayed in the target quadrant were recorded to assess the memory retrieval ability. The whole process was recorded by the Any‐maze tracking system.

### 2.9 Tetrazolium chloride (TTC) staining

Brains of P10 rats (3 days after HIBD model established) were harvested for TTC (2,3,5‐triphenyltetrazolium chloride) staining as previously described with modifications.^31^ Five coronal sections were taken for each brain. The incisions were at the middle of the forebrain and optic chiasma, optic chiasma, funnel stalk, and the midpoint of funnel stalk and caudal pole of posterior lobe. Slices were placed in 2% of TTC solution for 20 min at 37°C, flipped every 5 min, and followed by phosphate‐buffered saline (PBS) washing and photographing.^45^

### 2.10 Immunofluorescence

The immunofluorescence test was performed as previously described with modifications.^46^ Briefly, after behavioral tests, the rats were deeply anaesthetized and then transcardially perfused with PBS followed by 4% paraformaldehyde (PFA) in 100 mM PBS (PH 7.4). Then, the rats brains were collected and fixed in 4% PFA for 5 days at 4°C, followed by dehydration with 30% sucrose in 100 mM PBS for several days. Then, the brain was serially sectioned into 30‐μm coronal sections using a Leica cryostat and every 6 slices with the same reference position were stained.^47^ After blocking and permeabilization, the slices were incubated with diluted anti‐NeuN (1:200 dilution) overnight at 4°C, and on the next day incubated with secondary antibody Alex 568 (A11004, 1:1000 dilution, Invitrogen Life Technologies, Grand Island, NY, USA) for 2 h at room temperature (RT). Then, the sections were mounted with a DAPI included medium (Beyotime Biotechnology, Shanghai, China). The images were captured by a microscope (Leica Stellaris 5 WLL) at 20× magnification.

### 2.11 Enzyme‐linked immunosorbent assay (ELISA)

Brains from different groups were collected 48 h after the HIBD model was established, followed by two freeze–thaw cycles to break the brain cell membranes, and homogenization with tissue/saline rate of 1: 9 under the condition of an ice water bath. The lysates were then centrifuged at 5000 *g* for 5 min at 4°C. The supernatant was collected to measure the concentrations of IL‐6, IL‐1β, and TNF‐α using an ELISA Kit according to the manufacturer's instructions. The absorbance was determined at 450 nm by a microplate reader (Bio Tek Instruments) and the concentration of the target protein was calculated according to the standard curve and normalized against the protein of the samples. The results were expressed as pg/mL or ng/mL protein.

### 2.12 Western blotting

The total protein was extracted using a pre‐cooled whole cell lysis buffer. Approximately 40 μg of the total protein was boiled with 5 X sample buffers at 98°C for 5 min. Then, the samples were separated by 12.5% SDS‐PAGE gel electrophoresis and transferred onto an Immobilon‐PTM polyvinylidene difluoride (PVDF) membrane (#ISEQ00010, Millipore Corporation, Billerica, MA, USA). Then, the membrane was incubated with 5% fat‐free milk in PBS that contains 0.1% Tween‐20 for 90 min at RT to block the non‐specific binding site. The primary antibodies of the target proteins were incubated overnight at 4°C. The following day, the membrane was immunoblotted with corresponding horseradish peroxidase (HRP)‐conjugated secondary antibody (1: 7500, Perkin Elmer) for 90 min at RT. The chemiluminescence of the blots was visualized using a Syngene GBox Imaging System, and Bio‐Rad Quantity One software was used to measure the intensities of interested bands (raw data). Immunoblotting with anti‐β‐actin was used to control equal loading and protein quality.

### 2.13 Neuronal cell proliferation, cytotoxicity, and apoptosis assays

When the integrity of the cell's plasma membrane is compromised, lactate dehydrogenase (LDH) is released from the cells into the culture medium. The release of LDH in the neuron culture supernatant can represent the degree of cell death. In our study, the extracellular level of LDH was detected using an LDH cytotoxicity assay Kit. Firstly, we plated the neurons onto 96‐well plates (pre‐treated with poly‐D‐lysine‐coated) at a density of 1.0 × 10^5^ per well. Seven days later, the LDH level was detected according to the manufacturer's instructions. The LDH release reagent was used as a positive control to test the maximum LDH release. The absorbance was measured at 490 nm by a microplate reader (Bio Tek Instruments). The ratio (%) between the absorbance of the treatment group and that of the control group was used to represent the cell death rate.

Primary culture of hippocampal neurons (7 DIV) was washed with cold PBS, then fixed with 4% paraformaldehyde in 100 mM PBS for 20 min. Thereafter, the neurons were incubated with Annexin V‐FITC and propidium iodide (PI) for 30 min at RT by avoiding bright light. After sealing, a Nikon Eclipse Ti inverted fluorescence microscope was used to take the images. The cells that are Annexin V‐FITC positive and PI negative are considered to be in early apoptosis, while the cells that are both Annexin V‐FITC and PI positive are considered to be in late apoptosis or necrosis.

### 2.14 Image analysis

The infarct area measurement by TTC staining and the counting of NeuN+ cells were performed with Image J.

### 2.15 Statistical analysis

All data were expressed as means ± standard error (mean ± SEM). These differences of the rotarod test, spatial learning in the Morris water maze test, and TTC staining among different groups were analyzed using two‐way ANOVA with treatment (group) as the between‐subjects factor and trials as the within‐subjects factor. The data of all the other experiments were analyzed using one‐way ANOVA. All graphs were plotted as mean ± SEM with Prism 9.2.0 software. *p < 0.05, **p < 0.01, ***p < 0.001, ****p < 0.0001.

## 3 RESULTS

### 3.1 VX‐765 treatment ameliorates movement coordination and myodynamia deficits in HIBD rats

**FIGURE 1:**
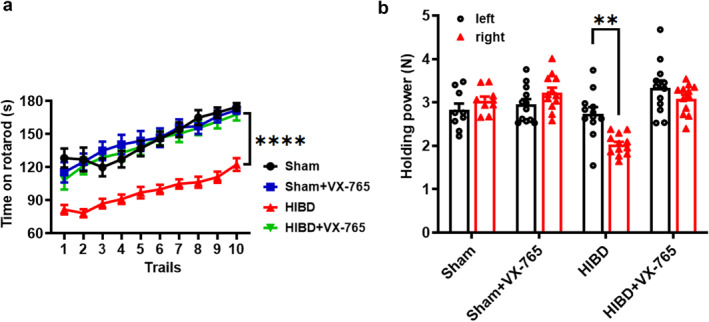
VX‐765 treatment ameliorates movement coordination and myodynamia deficits in HIBD rats. (A) VX‐765 treatment increases the time spent on the rod during the rotarod test in HIBD rats. (B) VX‐765 treatment increases holding power of right forelimb during the grasping test in HIBD rats. Data was presented as mean ± SEM. **p < 0.01, ****p < 0.0001.

It is reported that children with HIE experience neurodevelopment delay and motor ability deficits, and obviously the HIBD model rats do have these behavioral deficits.^39^, ^48^, ^49^ To confirm whether VX‐765 can rescue HIBD‐induced movement coordination and myodynamia deficits induced by HI, a rotarod test was used to evaluate the locomotor condition and the grasping test was used to evaluate the myodynamia. Rotarod test results showed that HIBD rats spent much less time on the rod compared to the rats in the Sham group (Sham: *n* = 10; HIBD: *n* = 10, *p* < 0.0001 vs. Sham; Figure 1A), indicating a significant deficit of movement coordination in HIBD rats. VX‐765 administration obviously rescued the HIBD‐induced movement coordination deficit, as reflected by a significant increase in time spent on the rod (HIBD + VX‐765: *n* = 10, *p* > 0.05 vs. Sham, *p* < 0.0001 vs. HIBD; Figure 1A), whereas VX‐765 treatment has no effect on movement coordination in the Sham group (Sham + VX‐765: *n* = 10, *p* > 0.05 vs. Sham; Figure 1A). In the grasping test, the right (HI side) forelimb myodynamia of rats was significantly lower than that of their left forelimb (negative control side) in the HIBD group (HIBD: *n* = 10, grip strength: left, 2.77 ± 0.18 N; right, 1.99 ± 0.08 N, *p* = 0.0011 vs. left; Figure 1B), rats of the other three groups did not have this effect (Sham: *n* = 10; Sham + VX‐765: *n* = 10, HIBD + VX‐765: *n* = 10; Figure 1B). These results suggest that VX‐765 treatment can alleviate the deficits of motor coordination and myodynamia in HIBD rats.

### 3.2 VX‐765 improves spatial learning and memory impairment in HIBD rats

**FIGURE 2:**
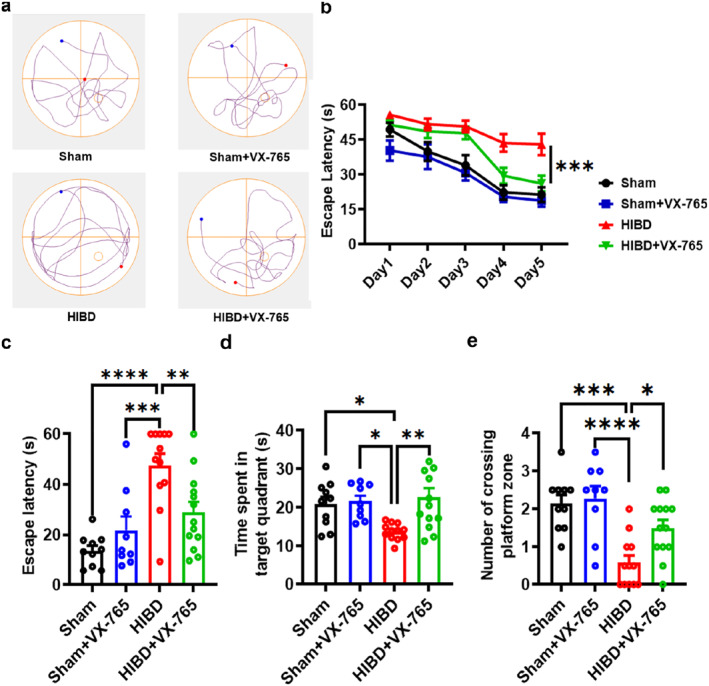
VX‐765 improves spatial learning and memory impairment in HIBD rats. (A) Representative track plot in the probe test in the Morris water maze test. (B) The escape latency to the hidden platform during spatial learning day. (C) The escape latency for finding the location of hidden platform. (D) The time spent in the original quadrant of the platform. (E) The number of crossing the location of hidden platform during the probe test. Data are expressed as mean ± SEM, *p < 0.05, **p < 0.01, ***p < 0.001, ****p < 0.001.

Focal cortical infarction of the HIBD model leads to secondary neuronal damage in the ipsilateral nonischemic hippocampus, which results in cognitive disorders.^31^ Here, the Morris water maze test was used to evaluate the effects of VX‐765 administration on spatial learning and memory impairments in neonatal rats subjected to HIBD. As shown in Figure 2, the rats in the HIBD group spent more time to find the hidden platform (the escape latency) than the rats in the Sham group during the training period (Sham: *n* = 10; Sham + VX‐765: *n* = 9, *p* > 0.05 vs. Sham; HIBD: *n* = 12, *p* < 0.0001 vs. Sham; Figure 2B), suggesting an impairment of spatial learning after HIBD. Treatment with VX‐765 significantly shortened the escape latency to find the hidden platform compared to the HIBD group rats (HIBD + VX‐765: *n* = 13, *p* = 0.0007 vs. HIBD; Figure 2B).

The escape latency, the time spent in the original quadrant of the platform and the number of crossing the location of hidden platform were recorded in probe test to assess spatial memory retrieval ability. As shown in Figure 2, the escape latency in the HIBD group was significantly longer than that in the Sham group (Sham: *n* = 10, 13.75 ± 2.02 s; Sham + VX‐765: *n* = 9, 21.88 ± 5.38 s, *p* > 0.05 vs. Sham; HIBD: *n* = 12, 47.70 ± 4.53 s, *p* < 0.0001 vs. Sham, *p* = 0.0009 vs. Sham + VX‐765; Figure 2C), suggesting that spatial memory retrieval was obviously impaired in HIBD rats. While, treatment with VX‐765 significantly shortened the escape latency in rats after HIBD (HIBD + VX‐765: *n* = 13, 28.85 ± 4.21 s, *p* = 0.0093 vs. HIBD; Figure 2C). In addition, compared to the Sham group rats, the HIBD group rats spent less time in the target quadrant within 60 s, while compared to the HIBD rats, VX‐765 administration obviously increased the time spent in the target quadrant (Sham: *n* = 10, 20.92 ± 1.87 s; Sham + VX‐765: *n* = 9, 21.61 ± 1.49 s, *p* > 0.05 vs. Sham; HIBD: *n* = 12, 13.75 ± 0.62 s, *p* = 0.0386 vs. Sham; HIBD + VX‐765: *n* = 13, 22.63 ± 2.42 s, *p* > 0.05 vs. Sham, *p* = 0.035 vs. HIBD; Figure 2D). Furthermore, more than half of the rats in the HIBD group could not find the destination when we counted the times of platform‐zone crossing (Sham: *n* = 10, 2.15 ± 0.22; Sham + VX‐765: *n* = 9, 2.28 ± 0.32, *p* > 0.05 vs. Sham; HIBD: *n* = 12, 0.58 ± 0.19, *p* = 0.0002 vs. Sham; HIBD + VX‐765: *n* = 13, 1.5 ± 0.21, *p* > 0.05 vs. Sham, *p* = 0.0257 vs. HIBD; Figure 2E). These results suggest that treatment with VX‐765 can improve the impairments of spatial learning and memory induced by HI.

### 3.3 VX‐765 decreases infarct volumes, neuron death and neuroinflammation in HIBD rats

**FIGURE 3:**
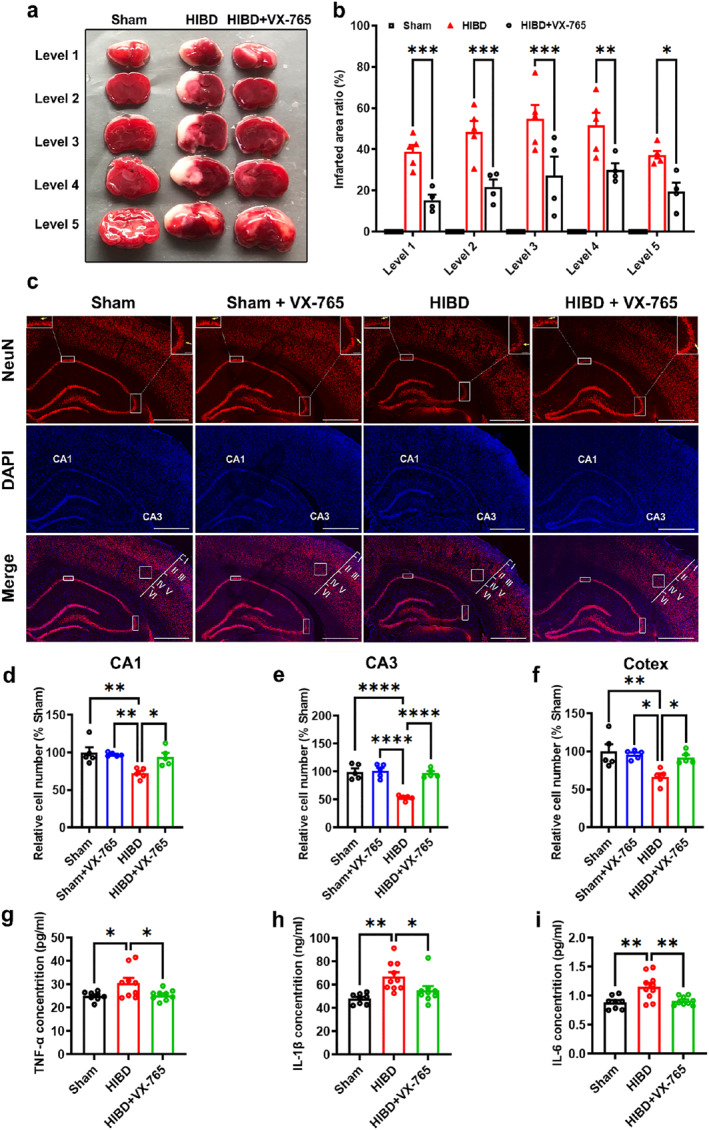
VX‐765 decreases infarct volumes, neuron death and neuroinflammation in HIBD rats. (A) Representative TTC staining images of the brain slices. Sections are labeled as five different levels (Level 1–Level 5) along the anterior to posterior axis. The white area indicates the liquefactive necrosis area. (B) Quantification of the cerebral infarct area in brain sections (infarcted area[%] = white [stainless] area/total area of the left side). (C) Representative immunofluorescence images of different groups. Images in the boxes at the top corner of NeuN channel: magnified images of each boxed area. Arrows in the magnified images indicate the NeuN+ cell loss in CA3/CA1 areas of hippocampus. The cortical area image in the box used to represent the count of cortical neurons. Scale bars = 1 mm (200 μm for the magnified images). (D–F) Bar graph summarizing the relative number of NeuN immunoreactive neurons in the CA1, CA3 areas of hippocampus and deeper layers of cortex. (G–I) VX‐765 reduced the HI‐induced brain proinflammatory cytokines TNF‐α (G), IL‐1β (H), and IL‐6 (I). Data were presented as mean ± SEM. *p < 0.05, **p < 0.01, ***p < 0.001, ****p < 0.0001. TTC, Tetrazolium chloride.

It is reported that liquefactive necrosis occurs when hypoxia and ischemia happen in brain.^28^ To further investigate the neuroprotective role of VX‐765 on brain injury in HIBD rats, TTC staining and immunofluorescence test was used. In TTC staining, compared with the Sham group, the cerebral infarct volume of rats in the HIBD group were significantly increased, while the cerebral infarct volume of rats treated with VX‐765 were evidently decreased (Sham: *n* = 4, HIBD: *n* = 5, HIBD + VX‐765: *n* = 4; Level 1: Sham, 0; HIBD, 38.69 ± 3.42%; HIBD + VX‐765, 15.31 ± 2.72%, *p* = 0.0009 vs. HIBD; Level 2: Sham, 0; HIBD, 48.52 ± 5.20%; HIBD + VX‐765, 21.71 ± 3.63%, *p* = 0.0002 vs. HIBD; Level 3: Sham, 0; HIBD, 54.83 ± 6.60%; HIBD + VX‐765, 27.30 ± 9.13%, *p* = 0.0001 vs. HIBD; Level 4: Sham, 0; HIBD, 51.61 ± 6.11%; HIBD + VX‐765, 30.06.68 ± 3.22%, *p* = 0.0023 vs. HIBD; Level 5: Sham, 0; HIBD, 37.20 ± 2.00%; HIBD + VX‐765, 19.50 ± 4.33%, *p* = 0.0141 vs. HIBD; Figure 3A,B). Immunofluorescence staining of NeuN was examined to determine the neuroprotective role of VX‐765 on hippocampal and cortex neurons in HIBD rats. As shown in Figure 3, the number of neurons in hippocampal and cortex region obviously decreased in the HIBD group rats than that in the Sham group rats, and this neuron number decrease could be obviously rescued by VX‐765 treatment (For CA1: Sham: *n* = 5; Sham + VX‐765: *n* = 5, 97.5 ± 1.10% Sham, *p* > 0.05 vs. Sham; HIBD: *n* = 5, 72.27 ± 3.12% Sham, *p* = 0.0039 vs. Sham, *p* = 0.0083 vs. Sham + VX‐765; HIBD + VX‐765: *n* = 5, 94.32 ± 5.33% Sham, *p* > 0.05 vs. Sham, *p* = 0.0216 vs. HIBD; Figure 3C,D; For CA3 area: Sham: *n* = 5; Sham + VX‐765: *n* = 5, 105.72 ± 5.40% Sham, *p* > 0.05 vs. Sham; HIBD: *n* = 5, 53.20 ± 2.04% Sham, *p* < 0.0001 vs. Sham, *p* < 0.0001 vs. Sham + VX765; HIBD + VX‐765: *n* = 5, 97.06 ± 3.48% Sham, *p* > 0.05 vs. Sham, *p* < 0.0001 vs. HIBD; Figure 3C,E; For the deeper layers of cortex [layer IV‐V]: Sham: *n* = 5; Sham + VX‐765: *n* = 5, 98.85 ± 2.61% Sham, *p* > 0.05 vs. Sham; HIBD: *n* = 5, 66.51 ± 4.58% Sham, *p* = 0.0043 vs. Sham, *p* = 0.0119 vs. Sham + VX765; HIBD + VX‐765: *n* = 5, 92.03 ± 3.69% Sham, *p* > 0.05 vs. Sham, *p* = 0.0304 vs. HIBD. Figure 3C,F). These results indicate that HI induced liquefactive necrosis and neuronal death could be significantly prevented by VX‐765 treatment.

Cerebral HI injury induced cell death leads to the release of damage‐associated molecular patterns (DAMPs). These DAMPs are known to trigger inflammatory events to induced the production of pro‐inflammatory cytokines, which in turn exacerbates cell death.^50^ To further explore the protective mechanism of VX‐765 on HIBD, we measured the expression of inflammation cytokines such as TNF‐α, IL‐1β, and IL‐6 by ELISA. The results showed that HI caused a significant increase in the secretion of TNF‐α, IL‐1β, and IL‐6. Notably, treatment with VX‐765 dramatically rescued the increase of TNF‐α, IL‐1β and IL‐6 induced by HIBD in the brain (For TNF‐α: Sham: *n* = 8, 25.07 ± 0.66; HIBD: *n* = 10, 30.62 ± 2.18, *p* = 0.0316 vs. Sham; HIBD + VX‐765: *n* = 9, 25.48 ± 0.58, *p* > 0.05 vs. Sham, *p* = 0.0413 vs. HIBD. Figure 3G; For IL‐1β: Sham: *n* = 8, 48.11 ± 1.58; HIBD: *n* = 10, 67.08 ± 3.89, *p* = 0.0019 vs. Sham; HIBD + VX‐765: *n* = 9, 55.16 ± 3.78, *p* > 0.05 vs. Sham, *p* = 0.0471 vs. HIBD; Figure 3H; For IL‐6: Sham: *n* = 8, 0.88 ± 0.04; HIBD: *n* = 10, 1.15 ± 0.070, *p* = 0.0034 vs. Sham; HIBD + VX‐765: *n* = 9, 0.911 ± 0.025, *p* > 0.05 vs. Sham, *p* = 0.0066 vs. HIBD; Figure 3I). Together, these results suggest that VX‐765 plays a protective role in HIBD by inhibiting HI induced cell death and neuroinflammation.

### 3.4 VX‐765 inhibits PANoptosis activation in HIBD rats

**FIGURE 4:**
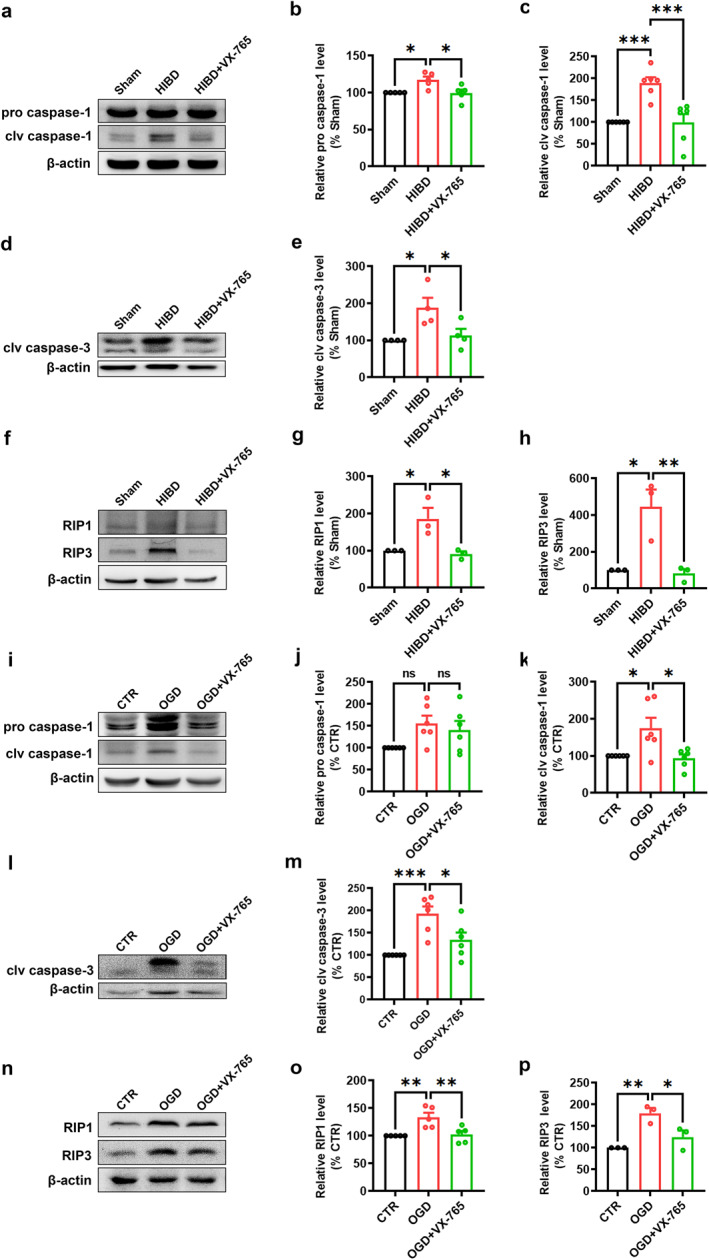
VX‐765 inhibits PANoptosis activation in HIBD rats. Western blot was used to detect the expression of the molecules after HI. Densitometry was used to measure the protein levels. (A–H) VX‐765 suppressed caspase‐1 activation induced by HI, and reduced HI‐induced high expression of clv caspase‐3, RIP1 and RIP3 in neonatal rats. (I–P) VX‐765 suppressed caspase‐1 activation induced by OGD, and reduced OGD‐induced high expression of clv caspase‐3, RIP1 and RIP3 in primary cultured neurons. The data was expressed as mean ± SEM. *p < 0.05, **p < 0.01, ***p < 0.001.

Since pyroptosis (caspase‐1), apoptosis (caspase‐3), and necroptosis (RIP1/RIP3) play a pivotal role in HIE, the cross‐talk of those three forms of cell death leads to the concept of PANoptosis, and caspase‐1 may be a critical component of the protein complex that initiates PANoptosis, we next detected the expression of PANoptosis to further determine the neuroprotective effect of VX‐765 in HI. Western blotting results revealed that the levels of caspase‐1, cleaved caspase‐3, and RIP1, RIP3 were significantly elevated in the HI group compared to the Sham group. Importantly, the heightened expression of these proteins induced by HI was substantially reduced by VX‐765 treatment (For pro caspase‐1: *n* = 5 in each group; HIBD: 117.18 ± 4.50% Sham, *p* = 0.0193 vs. Sham; HIBD + VX‐765: 99.39 ± 4.80% Sham, *p* > 0.05 vs. Sham, *p* = 0.0157 vs. HIBD; Figure 4A,B; For clv caspase‐1: *n* = 6 in each group; HIBD: 189.68 ± 13.02% Sham, *p* = 0.0007 vs. Sham; HIBD + VX‐765: 99.62 ± 19.03% Sham, *p* > 0.05 vs. Sham, *p* = 0.0007 vs. HIBD; Figure 4A,C; For clv caspase‐3: *n* = 4 in each group; HIBD: 187.64 ± 27.14% Sham, *p* = 0.0232 vs. Sham; HIBD + VX‐765: 113.00 ± 18.20% Sham, *p* > 0.05 vs. Sham, *p* = 0.0496 vs. HIBD; Figure 4D,E; For RIP1: *n* = 3 in each group; HIBD: 185.92 ± 29.56% Sham, *p* = 0.0314 vs. Sham; HIBD + VX‐765: 90.63 ± 7.42% Sham, *p* > 0.05 vs. Sham, *p* = 0.0203 vs. HIBD; Figure 4F,G; For RIP3: *n* = 3 in each group; HIBD: 445.67 ± 93.78% Sham, *p* = 0.0112 vs. Sham; HIBD + VX‐765: 81.75 ± 24.49% Sham, *p* > 0.05 vs. Sham, *p* = 0.0088 vs. HIBD; Figure 4F,H). Next, primarily cultured neurons treated with OGD/R to further examine the expression and activation of caspase‐1, cleaved caspase‐3 and RIP1, RIP3 in the present study. As expected, similar results were obtained in the primary neurons OGD/R model: cleaved caspase‐1, cleaved caspase‐3 and RIP1, RIP3 expression levels were markedly increased after OGD/R treatment, whereas VX‐765 reversed this effect (For pro caspase‐1: *n* = 6 in each group; OGD: 155.80 ± 17.64% CTR, *p* = 0.0569 vs. CTR; OGD + VX‐765: 140.80 ± 20.47% CTR, *p* > 0.05 vs. CTR, *p* = 0.779 vs. OGD; Figure 4I,J; For clv caspase‐1: *n* = 6 in each group; OGD: 174.63 ± 28.50% CTR, *p* = 0.0225 vs. CTR; OGD + VX‐765: 93.62 ± 10.45% CTR, *p* > 0.05 vs. CTR, *p* = 0.0135 vs. OGD; Figure 4I,K; For clv caspase‐3: *n* = 6 in each group; OGD: 192.61 ± 16.66% CTR, *p* = 0.0006 vs. CTR; OGD + VX‐765: 134.27 ± 16.54% CTR, *p* > 0.05 vs. CTR, *p* = 0.0211 vs. OGD; Figure 4l,M; For RIP1: *n* = 5 in each group; OGD: 133.37 ± 8.40% CTR, *p* = 0.0060 vs. CTR; OGD + VX‐765: 102.26 ± 6.47% CTR, *p* > 0.05 vs. CTR, *p* = 0.0096 vs. OGD; Figure 4N,O; For RIP3: *n* = 3 in each group; OGD: 179.87 ± 12.05% CTR, *p* = 0.0059 vs. CTR; OGD + VX‐765: 124.71 ± 15.45% CTR, *p* > 0.05 vs. CTR, *p* = 0.0316 vs. OGD; Figure 4N,P). Together, results in this part demonstrate that PANoptosis is activated after HI in vivo and in vitro, and VX‐765 can inhibit PANoptosis activation induced by HI.

### 3.5 VX‐765 reverses OGD induced neuronal injuries

**FIGURE 5:**
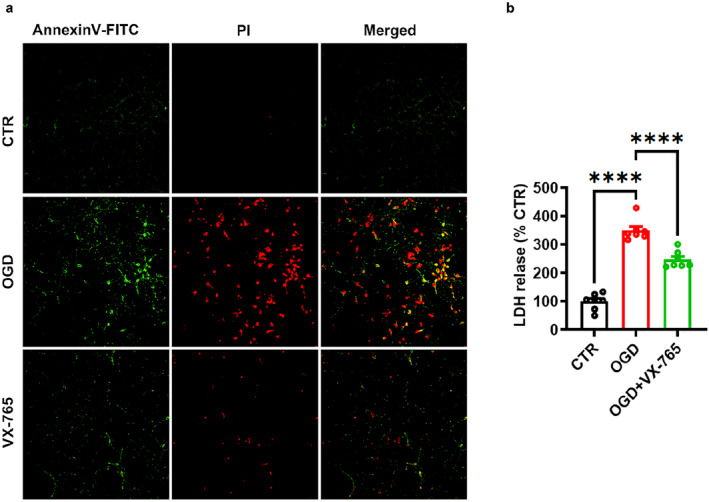
VX‐765 reverses OGD induced neuronal injuries. (A) Confocal images of annexin V‐FITC/PI stained the primary neurons. VX‐765 reduced the apoptosis and necrosis induced by OGD. (B) LDH assay revealed that VX‐765 prevented OGD‐induced excitotoxicity. Data was expressed as mean ± SEM. *p < 0.05, **p < 0.01, ****p < 0.0001.

Under conditions of HI stress, neuronal cells discharge DAMPs, leading to the expression of pro‐inflammatory mediators, and inflammation‐induced necrotic cell death.^51^ Our research using both in vitro and in vivo HI models demonstrated that VX‐765 could inhibit both apoptosis and necrosis. Furthermore, staining with annexin V‐FITC/PI revealed that OGD instigated apoptosis and necrosis in neurons, but this OGD‐induced heightened apoptosis and necrosis were mitigated by treatment with VX‐765 in primary cultured neurons (Figure 5A; Cells displaying Annexin V‐FITC positivity and PI negativity are identified as being in early apoptosis, while cells that are both Annexin V‐FITC and PI positive are considered to be in late apoptosis or necrosis). Cell death leads to disruption of the plasma membrane and cell lysis. LDH, a major cytoplasmic component, is released upon cell membrane disruption. Our findings revealed that HI induced membrane injury in neurons, as indicated by a significantly higher percentage of LDH release in the OGD‐treated group compared to the control group. However, treatment with VX‐765 significantly reduced LDH release induced by OGD (CTR: *n* = 7, OGD: *n* = 7, 350.27 ± 13.9% CTR, *p* < 0.0001 vs. CTR; OGD + VX‐765: *n* = 7, 247.29 ± 11.22% CTR, *p* < 0.0001 vs. OGD; Figure 5B). These findings indicate that VX‐765 can suppress cell death induced by OGD.

## 4 DISCUSSION

HIE is one of the main causes of neonatal neurological disease, which induces neurodevelopmental delays, neurological dysfunction, and memory impairments in children's development. But currently, there is no effective therapy method. Substantial preclinical evidence supports the view that inflammation, excitotoxicity, mitochondrial energy failure, and oxidative stress are the main pathophysiological factors causing perinatal cerebral HIBD.^38^, ^52^, ^53^ Brain injury caused by HI can persist for hours to weeks,^54^ early cerebral HI induced cell edema and dissolution.^55^ And a few minutes after HI immunity is triggered, leading to inflammatory cytokines release and cell death. Growing evidence has demonstrated that the caspase‐1 inflammasome signaling pathway is significantly activated in HI brain injuries.^25^, ^56^ Here, we found that VX‐765, the selective inhibitor of caspase‐1, suppresses neuron death by inhibiting neuroinflammation and the PANoptosis activation, and subsequently ameliorates motor coordination, myodynamia, spatial learning and memory impairment induced by HI in the neonatal rats.

Caspase‐1 is a key enzyme in the inflammatory response and is known to play a central role in pyroptosis, a form of programmed cell death associated with inflammation. Upon activation, Caspase‐1 cleaves gasdermin D, leading to the formation of pores in the cell membrane and subsequent cell lysis. This process is a hallmark of pyroptosis and is distinct from other forms of cell death such as apoptosis and necroptosis. Caspase‐1 has been associated with a range of neurological disorders in the CNS, including Stroke, Alzheimer's disease, Parkinson's disease, and multiple sclerosis.^57^, ^58^, ^59^ In these disorders, caspase‐1 serves as a crucial mediator of inflammatory responses, triggering neural cells to undergo various cell death paradigms, leading to nerve damage.^28^, ^31^ Studies have shown that HI increases the expression of caspase‐1 in both patients and animal models.^23^, ^25^ Our previous study using caspase‐1‐knockout mice has shown that genetic ablation of caspase‐1 improves HI‐induced myodynamia, motor coordination, learning and memory impairment in HIBD models.^28^ VX‐765 as a caspase‐1 inhibitor has been demonstrated in various inflammatory disease models by inhibiting pro‐caspase‐1 self‐cleavage.^58^ In the present study, we further found that VX‐765 in vivo markedly alleviate HI‐induced brain damage, as evidenced by VX‐765 administration significantly decreased infarction area, neuron death and neuroinflammation after HI, and improved long‐term neurobehavioral deficits induced by HI. It is known that both pyroptosis and necroptosis are inflammatory forms of programmed cell death which can release large amounts of proinflammatory cytokines. Caspase‐1 plays a significant role in promoting the release of cytokines, thus contributing to the inflammatory response and cell death. In our studies, we discovered VX‐765 effectively reduced the release of pro‐inflammatory cytokines such as TNF‐α, IL‐1β and IL‐6 induced by HI, thereby impeding the progression of inflammation in the CNS. These observations confirmed the neuroprotective role of VX‐765 against HI.

PANoptosis, a newly emerging concept, is a unique programmed cell death pathway that integrates elements of pyroptosis, apoptosis, and necroptosis. This concept underscores the cross‐talk and coordination between these three pathways.^28^, ^60^ It is a mechanism used by cells to respond to various physiological and pathological stimuli.^8^, ^9^, ^10^, ^11^, ^12^, ^61^ Subsequent studies showed redundant roles for caspase‐1/NLRP3, suggesting intersecting functions for pyroptotic, apoptotic, and necroptotic molecules.^17^, ^62^, ^63^, ^64^ In the context of our study, the increased expression of caspase‐1 suggests the activation of pyroptosis and potentially PANoptosis pathways. However, we acknowledge that demonstrating the activation of PANoptosis requires evidence of the concurrent activation of apoptosis and necroptosis pathways in addition to pyroptosis. To address this, we have conducted further experiments to assess the levels of apoptosis and necroptosis markers, such as caspase3, RIPK1/RIPK3. In our research, the use of VX‐765 effectively mitigated this elevated protein expression (clv caspase3, RIPK1/RIPK3), as shown in Figure 4. Furthermore, we found that in the Annexin V‐FITC/PI assay, the presence of Annexin V‐FITC positive and PI negative cells, representing early apoptosis, as well as both Annexin V‐FITC and PI positive cells, representing late apoptosis or necrosis, increased in the OGD models. Yet, the administration of VX‐765 was able to alleviate this manifestation, as shown in Figure 5. When HI occurs, the cell membrane ruptures and cell contents are released, which in turn induce cell death.^65^, ^66^, ^67^, ^68^ By In vitro we found that VX‐765 can reduce the cytoplasmic swelling (oncosis) and disruption by decreasing the release of LDH.

These results collectively suggest that VX‐765 can effectively attenuate inflammation and inhibit PANoptosis activation in the HIBD model, thereby improving neurological outcomes. These findings highlight the potential therapeutic value of VX‐765 in the treatment of HIBD and other inflammatory conditions associated with caspase‐1 action. However, further research is needed to fully understand the underlying mechanisms of VX‐765 in HIBD and to evaluate its safety and efficacy in clinical settings. Additionally, the optimal dosage, treatment duration, and potential effect sides of VX‐765 need to be determined through preclinical and clinical studies before it can be considered for therapeutic use in HI patients.

## 5 CONCLUSIONS

In summary, our present data suggest that the PANoptosis is activated after HI. VX‐765, the caspase‐1 specific inhibitor, prevents motor and spatial cognitive impairments induced by HIBD via reducing the cell death in vivo and in vitro through inhibiting PANoptosis activation by inhibiting the caspase‐1 function. These findings established a scientific foundation for the development of caspase‐1 inhibitors, VX‐765, as potential therapeutic interventions to improve the outcomes of HIBD patients.

## AUTHOR CONTRIBUTIONS

Xiaohuan Li, Chunfang Dai and Zhifang Dong conceived the study and wrote the manuscript. Xiaohuan Li, Boqing Xu and Mulan Chen performed behavioral studies. Xiaohuan Li, Chunfang Dai and Yepeng Fan performed biochemical assays. All authors read and approved the final manuscript.

## CONFLICT OF INTEREST STATEMENT

Zhifang Dong is a member of Pediatric Discovery Editorial Office. To minimize bias, he was excluded from all the editorial decision‐making to the acceptance of this article for publication. The other authors declare no competing interests.

## ETHICS STATEMENT

Animal ethics research was performed in accordance with the Chongqing Science and Technology Commission guidelines and approved by the Animal Ethics Committee of Children's Hospital of Chongqing Medical University (No. CHCMU‐IACUC20210114017).

## Data Availability

The data that support the findings of this study are available from the corresponding author upon reasonable request.

## References

[1] Kurinczuk JJ , White‐Koning M , Badawi N . Epidemiology of neonatal encephalopathy and hypoxic‐ischaemic encephalopathy. Early Hum Dev. 2010;86(6):329‐338. 20554402 10.1016/j.earlhumdev.2010.05.010

[2] Oza S , Lawn JE , Hogan DR , Mathers C , Cousens SN . Neonatal cause‐of‐death estimates for the early and late neonatal periods for 194 countries: 2000‐2013. Bull World Health Organ. 2015;93(1):19‐28. 25558104 10.2471/BLT.14.139790 PMC4271684

[3] Liu L , Oza S , Hogan D , et al. Global, regional, and national causes of child mortality in 2000‐13, with projections to inform post‐2015 priorities: an updated systematic analysis. Lancet. 2015;385(9966):430‐440. 25280870 10.1016/S0140-6736(14)61698-6

[4] Douglas‐Escobar M , Weiss MD . Hypoxic‐ischemic encephalopathy a review for the clinician. JAMA Pediatr. 2015;169(4):397‐403. 25685948 10.1001/jamapediatrics.2014.3269

[5] Liu CL , Siesjö BK , Hu BR . Pathogenesis of hippocampal neuronal death after hypoxia‐ischemia changes during brain development. Neuroscience. 2004;127(1):113‐123. 15219674 10.1016/j.neuroscience.2004.03.062 PMC3518049

[6] Hu BR , Liu CL , Ouyang Y , Blomgren K , Siesjö BK . Involvement of caspase‐3 in cell death after hypoxia‐ischemia declines during brain maturation. J Cerebr Blood Flow Metabol 2000;20(9):1294‐1300. 10.1097/00004647-200009000-00003 10994850

[7] Gluckman PD , Wyatt JS , Azzopardi D , et al. Selective head cooling with mild systemic hypothermia after neonatal encephalopathy: multicentre randomised trial. Lancet. 2005;365(9460):663‐670. 15721471 10.1016/S0140-6736(05)17946-X

[8] Man SM , Karki R , Kanneganti TD . Molecular mechanisms and functions of pyroptosis, inflammatory caspases and inflammasomes in infectious diseases. Immunol Rev. 2017;277(1):61‐75. 28462526 10.1111/imr.12534 PMC5416822

[9] Vanden Berghe T , Linkermann A , Jouan‐Lanhouet S , Walczak H , Vandenabeele P . Regulated necrosis: the expanding network of non‐apoptotic cell death pathways. Nat Rev Mol Cell Biol. 2014;15(2):134‐146. 10.1038/nrm3737 24452471

[10] Man SM , Kanneganti TD . Converging roles of caspases in inflammasome activation, cell death and innate immunity. Nat Rev Immunol. 2016;16(1):7‐21. 26655628 10.1038/nri.2015.7 PMC4915362

[11] Chan FKM , Luz NF , Moriwaki K . Programmed necrosis in the cross talk of cell death and inflammation. Annu Rev Immunol. 2015;33(1):79‐106. 25493335 10.1146/annurev-immunol-032414-112248 PMC4394030

[12] Blander JM . A long‐awaited merger of the pathways mediating host defence and programmed cell death. Nat Rev Immunol. 2014;14(9):601‐618. 25145756 10.1038/nri3720

[13] Christgen S , Zheng M , Kesavardhana S , et al. Identification of the PANoptosome: a molecular platform triggering pyroptosis, apoptosis, and necroptosis (PANoptosis). Front Cell Infect Microbiol. 2020;10:237. 32547960 10.3389/fcimb.2020.00237 PMC7274033

[14] Malireddi RKS , Gurung P , Kesavardhana S , et al. Innate immune priming in the absence of TAK1 drives RIPK1 kinase activity‐independent pyroptosis, apoptosis, necroptosis, and inflammatory disease. J Exp Med. 2020;217(3). 10.1084/jem.20191644 PMC7062518 31869420

[15] Lawlor KE , Khan N , Mildenhall A , et al. RIPK3 promotes cell death and NLRP3 inflammasome activation in the absence of MLKL. Nat Commun. 2015;6(1):6282. 25693118 10.1038/ncomms7282 PMC4346630

[16] Kuriakose T , Man SM , Malireddi RKS , et al. ZBP1/DAI is an innate sensor of influenza virus triggering the NLRP3 inflammasome and programmed cell death pathways. Sci Immunol. 2016;1(2):aag2045. 27917412 10.1126/sciimmunol.aag2045 PMC5131924

[17] Zheng M , Williams EP , Malireddi RKS , et al. Impaired NLRP3 inflammasome activation/pyroptosis leads to robust inflammatory cell death via caspase‐8/RIPK3 during coronavirus infection. J Biol Chem. 2020;295(41):14040‐14052. 32763970 10.1074/jbc.RA120.015036 PMC7549031

[18] Tsuchiya K . Switching from apoptosis to pyroptosis: gasdermin‐elicited inflammation and antitumor immunity. Int J Mol Sci. 2021;22(1):426. 33406603 10.3390/ijms22010426 PMC7794676

[19] Shi JJ , Zhao Y , Wang YP , et al. Inflammatory caspases are innate immune receptors for intracellular LPS. Nature. 2014;514(7521):187‐192. 25119034 10.1038/nature13683

[20] Wang YQ , Kanneganti TD . From pyroptosis, apoptosis and necroptosis to PANoptosis: a mechanistic compendium of programmed cell death pathways. Comput Struct Biotec. 2021;19:4641‐4657. 10.1016/j.csbj.2021.07.038 PMC8405902 34504660

[21] Heilig R , Dilucca M , Boucher D , et al. Caspase‐1 cleaves bid to release mitochondrial SMAC and drive secondary necrosis in the absence of GSDMD. Life Sci Alliance. 2020;3(6):e202000735. 32345661 10.26508/lsa.202000735 PMC7190276

[22] Ye XD , Song GN , Huang SS , et al. Caspase‐1: a promising target for preserving blood‐brain barrier integrity in acute stroke. Front Mol Neurosci. 2022;15:856372. 35370546 10.3389/fnmol.2022.856372 PMC8971909

[23] Wen S , Deng F , Li LL , Xu L , Li X , Fan QL . VX‐765 ameliorates renal injury and fibrosis in diabetes by regulating caspase‐1‐mediated pyroptosis and inflammation. J Diabetes Invest. 2022;13(1):22‐33. 10.1111/jdi.13660 PMC8756311 34494385

[24] Flores J , Noël A , Foveau B , Lynham J , Lecrux C , LeBlanc AC . Caspase‐1 inhibition alleviates cognitive impairment and neuropathology in an Alzheimer's disease mouse model. Nat Commun. 2018;9(1):3916. 30254377 10.1038/s41467-018-06449-x PMC6156230

[25] Hou XW , Yuan ZJ , Wang X , Cheng R , Zhou XG , Qiu J . Peptidome analysis of cerebrospinal fluid in neonates with hypoxic‐ischemic brain damage. Mol Brain. 2020;13(1):133. 33008433 10.1186/s13041-020-00671-9 PMC7531121

[26] Chen G , Shaw MH , Kim YG , Nuñez G . NOD‐like receptors: role in innate immunity and inflammatory disease. Annu Rev Pathol Mech. 2009;4(1):365‐398. 10.1146/annurev.pathol.4.110807.092239 18928408

[27] Martinon F , Burns K , Tschopp J . The inflammasome: a molecular platform triggering activation of inflammatory caspases and processing of proIL‐β. Mol Cell 2002;10(2):417‐426. 12191486 10.1016/s1097-2765(02)00599-3

[28] Chen YX , Li XH , Xiong Q , et al. Inhibiting NLRP3 inflammasome signaling pathway promotes neurological recovery following hypoxic‐ischemic brain damage by increasing p97‐mediated surface GluA1‐containing AMPA receptors. J Transl Med. 2023;21(1):567. 37620837 10.1186/s12967-023-04452-5 PMC10463885

[29] Zhang WH , Wang X , Narayanan M , et al. Fundamental role of the Rip2/caspase‐1 pathway in hypoxia and ischemia‐induced neuronal cell death. Proc Natl Acad Sci USA. 2003;100(26):16012‐16017. 14663141 10.1073/pnas.2534856100 PMC307684

[30] Zhang YT , Yao ZH , Xiao Y , Zhang XL , Liu JX . Downregulated XBP‐1 rescues cerebral ischemia/reperfusion injury‐induced pyroptosis via the NLRP3/caspase‐1/GSDMD Axis. Mediat Inflamm. 2022;2022:8007078. 10.1155/2022/8007078 PMC9050284 35497095

[31] Bellut M , Papp L , Bieber M , Kraft P , Stoll G , Schuhmann MK . NLPR3 inflammasome inhibition alleviates hypoxic endothelial cell death in vitro and protects blood‐brain barrier integrity in murine stroke. Cell Death Dis. 2022;13(1):20. 10.1038/s41419-021-04379-z PMC8688414 34930895

[32] Stack JH , Beaumont K , Larsen PD , et al. IL‐converting enzyme/caspase‐I inhibitor VX‐765 blocks the hypersensitive response to an inflammatory stimulus in monocytes from familial cold autoinflammatory syndrome patients. J Immunol 2005;175(4):2630‐2634. 16081838 10.4049/jimmunol.175.4.2630

[33] Wannamaker W , Davies R , Namchuk M , et al. (S)‐1‐((S)‐2‐{[1‐(4‐amino‐3‐chloro‐phenyl)‐methanoyl]‐amino}‐3,3‐dimethyl‐butanoyl)‐pyrrolidine‐2‐carboxylic acid ((2R,3S)‐2‐ethoxy‐5‐oxo‐tetrahydro‐furan‐3‐yl)‐amide (VX‐765), an orally available selective interleukin (IL)‐converting enzyme/caspase‐1 inhibitor, exhibits potent anti‐inflammatory activities by inhibiting the release of IL‐1β and IL‐18. J Pharmacol Exp Therapeut. 2007;321(2):509‐516. 10.1124/jpet.106.111344 17289835

[34] Liu X , Zhang ZB , Ruan JB , et al. Inflammasome‐activated gasdermin D causes pyroptosis by forming membrane pores. Nature. 2016;535(7610):153‐158. 27383986 10.1038/nature18629 PMC5539988

[35] Mula M . Emerging drugs for focal epilepsy. Expet Opin Emerg Drugs. 2013;18(1):87‐95. 10.1517/14728214.2013.750294 23176519

[36] Li J , Hao JH , Yao D , et al. Caspase‐1 inhibition prevents neuronal death by targeting the canonical inflammasome pathway of pyroptosis in a murine model of cerebral ischemia. Cns Neurosci Ther. 2020;26(9):925‐939. 32343048 10.1111/cns.13384 PMC7415206

[37] Qin ZJ , Song JQ , Lin AL , et al. GPR120 modulates epileptic seizure and neuroinflammation mediated by NLRP3 inflammasome. J Neuroinflammation. 2022;19(1):121. 35624482 10.1186/s12974-022-02482-2 PMC9137133

[38] Liang YB , Song PP , Chen W , et al. Inhibition of caspase‐1 ameliorates ischemia‐associated blood‐brain barrier dysfunction and integrity by suppressing pyroptosis activation. Front Cell Neurosci. 2021;14:540669. 33584203 10.3389/fncel.2020.540669 PMC7874210

[39] Dai CF , Wu B , Chen YX , et al. Aagab acts as a novel regulator of NEDD4‐1‐mediated PTEN nuclear translocation to promote neurological recovery following hypoxic‐ischemic brain damage. Cell Death Differ. 2021;28(8):2367‐2384. 33712741 10.1038/s41418-021-00757-4 PMC8328997

[40] Zhang S , Taghibiglou C , Girling K , et al. Critical role of increased PTEN nuclear translocation in excitotoxic and ischemic neuronal injuries. J Neurosci. 2013;33(18):7997‐8008. 23637190 10.1523/JNEUROSCI.5661-12.2013 PMC6618960

[41] Yang DX , Qiu J , Zhou HH , et al. Dihydroartemisinin alleviates oxidative stress in bleomycin‐induced pulmonary fibrosis. Life Sci. 2018;205:176‐183. 29752961 10.1016/j.lfs.2018.05.022

[42] Tang M , Wang RY , Feng PP , et al. Dihydroartemisinin attenuates pulmonary hypertension through inhibition of pulmonary vascular remodeling in rats. J Cardiovasc Pharmacol. 2020;76(3):337‐348. 32569012 10.1097/FJC.0000000000000862

[43] Yang DX , Yuan WD , Lv CJ , et al. Dihydroartemisinin supresses inflammation and fibrosis in bleomycine‐induced pulmonary fibrosis in rats. Int J Clin Exp Pathol. 2015;8(2):1270‐1281. 25973011 PMC4396330

[44] Vorhees CV , Williams MT . Morris water maze: procedures for assessing spatial and related forms of learning and memory. Nat Protoc. 2006;1(2):848‐858. 17406317 10.1038/nprot.2006.116 PMC2895266

[45] Benedek A , Móricz K , Jurányi Z , et al. Use of TTC staining for the evaluation of tissue injury in the early phases of reperfusion after focal cerebral ischemia in rats. Brain Res. 2006;1116(1):159‐165. 16952339 10.1016/j.brainres.2006.07.123

[46] Dai CF , Liu YM , Dong ZF . Tanshinone I alleviates motor and cognitive impairments via suppressing oxidative stress in the neonatal rats after hypoxic‐ischemic brain damage. Mol Brain. 2017;10(1):52. 29137683 10.1186/s13041-017-0332-9 PMC5686905

[47] Shi XY , Lim YS , Myers AK , et al. PIK3R2/Pik3r2 activating mutations result in brain overgrowth and EEG changes. Ann Neurol. 2020;88(6):1077‐1094. 32856318 10.1002/ana.25890 PMC8176885

[48] van Schie PEM , Schijns J , Becher JG , Barkhof F , van Weissenbruch MM , Vermeulen RJ . Long‐term motor and behavioral outcome after perinatal hypoxic‐ischemic encephalopathy. Eur J Paediatr Neurol. 2015;19(3):354‐359. 25683783 10.1016/j.ejpn.2015.01.005

[49] Erdi‐Krausz G , Rocha R , Brown A , et al. Neonatal hypoxic‐ischaemic encephalopathy: motor impairment beyond cerebral palsy. Eur J Paediatr Neurol. 2021;35:74‐81. 34666231 10.1016/j.ejpn.2021.10.005

[50] Weinstein JR , Koerner IP , Moller T . Microglia in ischemic brain injury. Future Neurol. 2010;5(2):227‐246. 20401171 10.2217/fnl.10.1 PMC2853969

[51] Iadecola C , Anrather J . The immunology of stroke: from mechanisms to translation. Nat Med. 2011;17(7):796‐808. 21738161 10.1038/nm.2399 PMC3137275

[52] Ravizza T , Noé F , Zardoni D , Vaghi V , Sifringer M , Vezzani A . Interleukin converting enzyme inhibition impairs kindling epileptogenesis in rats by blocking astrocytic IL‐1β production. Neurobiol Dis. 2008;31(3):327‐333. 18632279 10.1016/j.nbd.2008.05.007

[53] Yuan JY , Amin P , Ofengeim D . Necroptosis and RIPK1‐mediated neuroinflammation in CNS diseases. Nat Rev Neurosci. 2019;20(1):19‐33. 30467385 10.1038/s41583-018-0093-1 PMC6342007

[54] McKinstry RC , Miller JH , Snyder AZ , et al. A prospective, longitudinal diffusion tensor imaging study of brain injury in newborns. Neurology. 2002;59(6):824‐833. 12297561 10.1212/wnl.59.6.824

[55] Yang JP , Zhao YY , Zhang L , et al. RIPK3/MLKL‐Mediated neuronal necroptosis modulates the M1/M2 polarization of microglia/macrophages in the ischemic cortex. Cerebr Cortex. 2018;28(7):2622‐2635. 10.1093/cercor/bhy089 PMC5998990 29746630

[56] Shan B , Pan HL , Najafov A , Yuan JY . Necroptosis in development and diseases. Gene Dev. 2018;32(5‐6):327‐340. 29593066 10.1101/gad.312561.118 PMC5900707

[57] Gong Z , Pan JR , Shen QY , Li M , Peng Y . Mitochondrial dysfunction induces NLRP3 inflammasome activation during cerebral ischemia/reperfusion injury. J Neuroinflammation. 2018;15(1):242. 30153825 10.1186/s12974-018-1282-6 PMC6114292

[58] Chen J , Chen YQ , Shi YJ , et al. VX‐765 reduces neuroinflammation after spinal cord injury in mice. Neural Regen Res. 2021;16(9):1836‐1847. 33510091 10.4103/1673-5374.306096 PMC8328782

[59] Zhang XH , Li ML , Wang B , Guo MX , Zhu RM . Caspase‐1 inhibition alleviates acute renal injury in rats with severe acute pancreatitis. World J Gastroenterol. 2014;20(30):10457‐10463. 25132762 10.3748/wjg.v20.i30.10457 PMC4130853

[60] Samir P , Malireddi RKS , Kanneganti TD . The PANoptosome: a deadly protein complex driving pyroptosis, apoptosis, and necroptosis (PANoptosis). Front Cell Infect Microbiol. 2020;10:238. 32582562 10.3389/fcimb.2020.00238 PMC7283380

[61] Zheng M , Kanneganti TD . The regulation of the ZBP1‐NLRP3 inflammasome and its implications in pyroptosis, apoptosis, and necroptosis (PANoptosis). Immunol Rev. 2020;297(1):26‐38. 32729116 10.1111/imr.12909 PMC7811275

[62] Gurung P , Burton A , Kanneganti TD . NLRP3 inflammasome plays a redundant role with caspase 8 to promote IL‐1β‐mediated osteomyelitis. Proc Natl Acad Sci USA. 2016;113(16):4452‐4457. 27071119 10.1073/pnas.1601636113 PMC4843439

[63] Lukens JR , Gurung P , Vogel P , et al. Dietary modulation of the microbiome affects autoinflammatory disease. Nature. 2014;516(7530):246‐249. 25274309 10.1038/nature13788 PMC4268032

[64] Tsuchiya K , Nakajima S , Hosojima S , et al. Caspase‐1 initiates apoptosis in the absence of gasdermin D. Nat Commun. 2019;10(1):2091. 31064994 10.1038/s41467-019-09753-2 PMC6505044

[65] Sedgwick JD , Riminton DS , Cyster JG , Körner H . Tumor necrosis factor: a master‐regulator of leukocyte movement. Immunol Today 2000;21(3):110‐113. 10689296 10.1016/s0167-5699(99)01573-x

[66] Sriram K , Matheson JM , Benkovic SA , Miller DB , Luster MI , O'Callaghan JP . Deficiency of TNF receptors suppresses microglial activation and alters the susceptibility of brain regions to MPTP‐induced neurotoxicity: role of TNF‐α. FASEB J. 2006;20(6):670‐682. 16581975 10.1096/fj.05-5106com

[67] Rothwell N . Interleukin‐1 and neuronal injury: mechanisms, modification, and therapeutic potential. Brain Behav Immun. 2003;17(3):152‐157. 12706413 10.1016/s0889-1591(02)00098-3

[68] Li J , Zhang JY , Zhang YS , et al. TRAF2 protects against cerebral ischemia‐induced brain injury by suppressing necroptosis. Cell Death Dis. 2019;10(5):328. 30988281 10.1038/s41419-019-1558-5 PMC6465397

